# Quercetin nanoparticles exhibit a potential therapeutic effect against the neurochemical changes and motor deficits in a rat model of Parkinson’s disease

**DOI:** 10.1038/s41598-026-63820-5

**Published:** 2026-08-05

**Authors:** Hend El-bakry, Yasser A. Khadrawy, Ibrahim H. Ibrahim, Haitham S. Mohammed

**Affiliations:** 1 Department of Physics, Faculty of Science, Ain-Shams University, Cairo, Egypt; 2 Medical Physiology Department, National Research Center, Giza, Egypt; 3 Biophysics Department, Faculty of Science, Cairo University, Giza, Egypt

**Keywords:** PD, Quercetin, Monoamines, Neuroenzymes, Oxidative stress, Rats, Biochemistry, Drug discovery, Neurology, Neuroscience

## Abstract

The present study investigated the therapeutic potential of quercetin nanoparticles (QNPs), administered alone or in combination with levodopa/carbidopa (L/C), against reserpine-induced neurochemical alterations and motor dysfunction in a rat model of Parkinson’s disease (PD). Animals were randomly assigned to five experimental groups: a control group, a reserpine-induced PD group, and PD groups treated with QNPs, L/C, or their combination. Motor performance was assessed using the open-field and grid traction tests. Reserpine administration induced marked motor impairments accompanied by significant reductions in dopamine (DA), serotonin (5-HT), and norepinephrine (NE) levels in both the striatum and midbrain. These neurochemical deficits were associated with elevated monoamine oxidase (MAO) activity, increased lipid peroxidation (malondialdehyde, MDA), enhanced acetylcholinesterase (AChE) activity, and increased nitric oxide (NO) levels in both brain regions. Additionally, reduced glutathione (GSH) levels were decreased in the midbrain but elevated in the striatum. Treatment with QNPs significantly restored monoamine levels and normalized MAO and AChE activities. Moreover, QNPs markedly attenuated oxidative stress and improved motor performance in reserpine-treated rats. Similarly, L/C administration effectively reversed monoamine depletion, normalized enzymatic activities, and mitigated oxidative stress. Notably, combined treatment with QNPs and L/C produced effects comparable to those observed with either treatment alone, suggesting a lack of additive or synergistic interaction. These findings demonstrate that QNPs exert a significant neuroprotective effect against reserpine-induced motor dysfunction and neurochemical disturbances, supporting their potential as a therapeutic strategy for Parkinsonian pathology.

## Introduction

Parkinson’s disease (PD) is a progressive neurodegenerative disorder characterized by the selective loss of dopaminergic neurons in the substantia nigra pars compacta (SNc), leading to debilitating motor and non-motor impairments. Despite decades of intensive investigation, the precise mechanisms underlying dopaminergic neuronal degeneration remain incompletely understood. Accumulating evidence suggests that PD pathogenesis is multifactorial, involving aging, mitochondrial dysfunction, oxidative stress, neuroinflammation, impaired protein degradation, and dysregulated autophagy^1–3^.

Oxidative stress is considered a central contributor to PD pathophysiology. Excessive production of reactive oxygen species (ROS), excitotoxic neuronal injury, mitochondrial impairment, and inflammation-mediated cytotoxicity collectively promote oxidative damage to dopaminergic neurons within the SNc^4^. The high metabolic demands of neurons, together with dopamine auto-oxidation and reduced antioxidant defenses, render these neurons particularly vulnerable to oxidative injury^5,6^.

Dopamine synthesized in the SNc plays a pivotal role in regulating motor coordination and non-motor functions. Progressive degeneration of SNc dopaminergic neurons represents the neuropathological hallmark of PD and underlies the characteristic motor symptoms, including bradykinesia, rigidity, and tremor, as well as non-motor manifestations^7,8^. Neurotrophic factors, particularly brain-derived neurotrophic factor (BDNF), are critical for synaptic plasticity, cognitive function, and dopaminergic neuron survival, and their dysregulation has been implicated in PD progression^9,10^.

PD is the second most prevalent neurodegenerative disorder after Alzheimer’s disease (AD), affecting approximately 0.2% of the global population. Its prevalence increases to nearly 1% in individuals over 60 years of age and rises sharply to about 4% in those over 80 years^11,12^. Current pharmacological strategies primarily focus on symptomatic relief through restoration of striatal dopamine levels, without halting disease progression (LeWitt, 2015). Levodopa (L-DOPA), administered in combination with carbidopa (L/C), remains the gold-standard therapy; however, long-term use is associated with declining efficacy and significant adverse effects, including motor fluctuations and dyskinesias^13^.

Consequently, there is increasing interest in identifying neuroprotective agents capable of targeting the underlying pathogenic mechanisms of PD. Quercetin (QC), a naturally occurring flavonoid, has attracted considerable attention due to its potent antioxidant and anti-inflammatory properties^14^. Quercetin exerts neuroprotective effects primarily by scavenging ROS and attenuating oxidative damage, a major contributor to dopaminergic neuronal degeneration in PD^15^. However, the clinical translation of quercetin is severely limited by its poor oral bioavailability (< 10%), largely attributed to its hydrophobic nature and rapid metabolism^16^.

Nanoparticle-based delivery systems have emerged as a promising strategy to enhance the bioavailability and therapeutic efficacy of poorly soluble phytochemicals. Therefore, the present study was designed to investigate the neuroprotective effects of quercetin nanoparticles (QNPs), administered alone or in combination with levodopa/carbidopa, on neurochemical alterations and motor deficits induced by reserpine in a rat model of PD. We hypothesized that QNPs would attenuate reserpine-induced motor deficits and neurochemical disturbances by reducing oxidative stress and restoring monoaminergic neurotransmission, and that their effects would be comparable to, or potentially enhance, those of levodopa/carbidopa.

## Materials and methods

### Chemicals

Quercetin and reserpine were purchased from Sigma-Aldrich (St. Louis, MO, USA). Levodopa/carbidopa (L/C) was obtained in its commercial formulation (Sinemet^®^, MSD, USA). All other chemicals and reagents used in the study were of analytical grade.

### Experimental animals

Adult male Wistar albino rats (200–250 g) were obtained from a certified local animal supplier. Animals were housed in plastic cages with stainless steel wire lids (eight rats per cage) and maintained under controlled environmental conditions (temperature: 25 ± 2 °C; relative humidity: 30–40%) with a 12 h light/12 h dark cycle. Rats were acclimatized for two weeks prior to the experiment and had free access to standard laboratory chow and water throughout the study.

All experimental procedures were conducted in accordance with institutional and international guidelines for the care and use of laboratory animals and were approved by the Biological Science Research Ethics Committee (ASU-SCI/ZOOL/2025/9/8) and the Natural and Earth Research Ethics Committee (ASU-SCI/PHYS/2025/07/01). The experimental animals were sacrificed by rapid decapitation without prior anesthesia to enable immediate tissue collection and to avoid anesthetic-induced alterations in neurochemical and biochemical parameters.

### Preparation of quercetin nanoparticles (QNPs)

Quercetin nanoparticles were prepared using the evaporative precipitation of nanosuspension (EPN) method as previously described^17^, with minor modifications^18^. Briefly, quercetin was dissolved in 99% ethanol at a concentration of 5 mg/mL and loaded into a syringe pump. The solution was injected into deionized water at a solvent-to-antisolvent ratio of 1:35 under constant magnetic stirring (1000 rpm) at a fixed flow rate of 8 mL/min. The resulting nanosuspension was filtered and vacuum-dried to obtain quercetin nanoparticles.

### Characterization of quercetin nanoparticles

#### Dynamic light scattering (DLS) and zeta potential

Particle size distribution, polydispersity index (PDI), and zeta potential of QNPs were determined using a Zetasizer Nano ZN (Malvern Panalytical Ltd., UK). Samples were sonicated for 5 min using a probe sonicator and diluted tenfold with deionized water prior to analysis. Measurements were performed at 25 °C using a fixed scattering angle of 173°. All measurements were conducted in triplicate.

#### Transmission electron microscopy (TEM)

The morphology and size of QNPs were examined by transmission electron microscopy (TEM; JEOL JEM-2100, Japan) operating at 100 kV. A drop of diluted QNP suspension was negatively stained with 1% uranyl acetate, placed onto a carbon-coated copper grid, air-dried, and subsequently examined under the TEM.

#### Fourier transform infrared (FTIR) spectroscopy

FTIR analysis was performed using a Vertex high-performance FTIR spectrometer (Bruker, Germany) equipped with an attenuated total reflectance (ATR) unit. Spectra were recorded over a wavelength range of 400–7500 cm⁻¹ at a spectral resolution of 16 cm⁻¹ with a minimum of 50 scans per sample.

#### Induction of the PD model

Parkinsonism was induced using reserpine as previously reported^19^. Reserpine was administered intraperitoneally at a dose of 0.02 mg/kg/day for 25 consecutive days. On day 26, motor activity was assessed to confirm PD induction. A maintenance dose of reserpine (0.01 mg/kg/day, i.p.) was administered for an additional 21 days to sustain Parkinsonian symptoms.

#### Experimental design

Animals were randomly assigned into two main groups: a control group receiving intraperitoneal injections of normal saline (0.9%) for 8 weeks, and a PD group subjected to reserpine administration. The PD group was further subdivided into four experimental groups: (i) untreated PD model group; (ii) PD + quercetin nanoparticles (50 mg/kg/day); (iii) PD + levodopa/carbidopa (25 mg/kg/day); and (iv) PD + combined quercetin nanoparticles (50 mg/kg/day) and levodopa/carbidopa (25 mg/kg/day). Treatments were administered daily throughout the experimental period.

Motor activity was evaluated in all groups prior to sacrifice. At the end of the 47-day experimental period, rats were euthanized, and brains were rapidly removed and bisected along the midline. Each hemisphere was dissected on ice into the midbrain and striatum.

The right hemispheres were used for the determination of monoamine neurotransmitter levels (dopamine, serotonin, and norepinephrine), whereas the left hemispheres were used for the assessment of oxidative stress markers, acetylcholinesterase (AChE) and monoamine oxidase (MAO) activities, brain-derived neurotrophic factor (BDNF), and tumor necrosis factor-α (TNF-α).

### Behavioral assessments

#### Open field test (OFT)

Locomotor activity and exploratory behavior were assessed using the open field test as previously described^20^. The apparatus consisted of a white plywood arena (72 × 72 cm) enclosed by 36-cm-high walls. The floor was divided into 16 equal peripheral squares and one central square (18 × 18 cm). Each rat was individually placed in the center of the arena and allowed to explore freely for 10 min. Behavioral activity was recorded using a video camera positioned above the apparatus. The following parameters were quantified: time spent in the central square, number of line crossings (defined as crossing grid lines with all four paws), number of rearings (standing on hind limbs), and freezing time (complete immobility). To eliminate olfactory cues, the apparatus was cleaned with 70% ethanol and allowed to dry between trials.

#### Grip strength test

Forelimb muscle strength was evaluated using the grip strength test, a validated method for assessing neuromuscular function and motor deficits^21^. Each rat was gently held by the tail and allowed to grasp a trapeze bar with its forepaws. The rat was then steadily pulled backward until it released the bar. The maximal force exerted before release was recorded in kilograms-force (kgf). Body weight was measured prior to testing to account for weight-related variability. Each rat underwent three consecutive trials, and the mean value was calculated for statistical analysis.

### Neurochemical and biochemical analyses

#### Determination of monoamine neurotransmitters

Levels of dopamine (DA), serotonin (5-HT), and norepinephrine (NE) in the midbrain and striatum were determined using the spectrofluorometric method described by Ciarlone^22^. Tissue samples were homogenized in chilled acidified n-butanol and centrifuged at 3000 rpm for 5 min. An aliquot of the supernatant (2.5 mL) was mixed with n-heptane (5 mL) and 0.2 N acetic acid (1.6 mL), vortexed, and centrifuged at 3000 rpm for 5 min. The organic phase was discarded, and the aqueous phase was collected for analysis. Fluorescence measurements were performed using a spectrofluorometer (Jasco FP-6500, JASCO Ltd., Tokyo, Japan) equipped with a 150-W xenon arc lamp, with excitation and emission slit widths set at 5 nm.

#### Tissue preparation for biochemical assays

The midbrain and striatum from the left hemisphere were homogenized in ice-cold Tris–HCl buffer (pH 7.4). Homogenates were centrifuged at 4000 rpm for 15 min at 4 °C. The resulting supernatants were used for the assessment of oxidative stress markers, enzyme activities, and cytokine levels.

### Oxidative stress parameters

#### Lipid peroxidation (malondialdehyde, MDA)

Lipid peroxidation was estimated by measuring malondialdehyde (MDA) levels according to the method of Ruiz-Larrea et al.^23^, based on the thiobarbituric acid reactive substances (TBARS) assay. The formation of a pink chromogen following reaction with thiobarbituric acid was measured spectrophotometrically at 532 nm.

#### Nitric oxide (NO)

Nitric oxide levels were determined indirectly by measuring nitrite concentrations using the Griess reaction, as described by Moshage et al.^24^. The resulting azo dye was quantified spectrophotometrically at 450 nm.

#### Reduced glutathione (GSH)

Reduced glutathione levels were measured using the method of Moron et al.^25^. This assay is based on the reduction of Ellman’s reagent (DTNB) by sulfhydryl groups, producing a yellow-colored compound measured at 412 nm.

### Enzyme activity assays

#### Acetylcholinesterase (AChE)

AChE activity was determined according to the method of Gorun et al.^26^. The enzymatic hydrolysis of acetylthiocholine iodide generates thiocholine, which reacts with DTNB to produce a yellow-colored anion. Absorbance was measured at 412 nm.

#### Monoamine oxidase (MAO)

MAO activity was assessed using the method described by Holt et al.^27^, based on the enzymatic oxidation of benzylamine to benzaldehyde. Absorbance was measured spectrophotometrically at 280 nm.

### Determination of BDNF and TNF-α

Brain-derived neurotrophic factor (BDNF) and tumor necrosis factor-α (TNF-α) levels in the midbrain and striatum were quantified using commercially available ELISA kits (Biokit for Scientific Research), according to the manufacturers’ instructions.

### Statistical analysis

Data are presented as mean ± standard error of the mean (SEM). Statistical analyses were performed using SPSS software (version 14.0). Group comparisons were conducted using one-way analysis of variance (ANOVA), followed by Duncan’s multiple range test as a post hoc analysis when appropriate. Duncan’s post hoc test was selected because it provides adequate sensitivity for detecting intergroup differences in exploratory biological studies involving multiple treatment conditions. Data normality and homogeneity of variance were verified before parametric analysis. Differences were considered statistically significant at *p* < 0.05.

## Results

### Characterization of quercetin nanoparticles

#### Particle size distribution and zeta potential

**Fig. 1:**
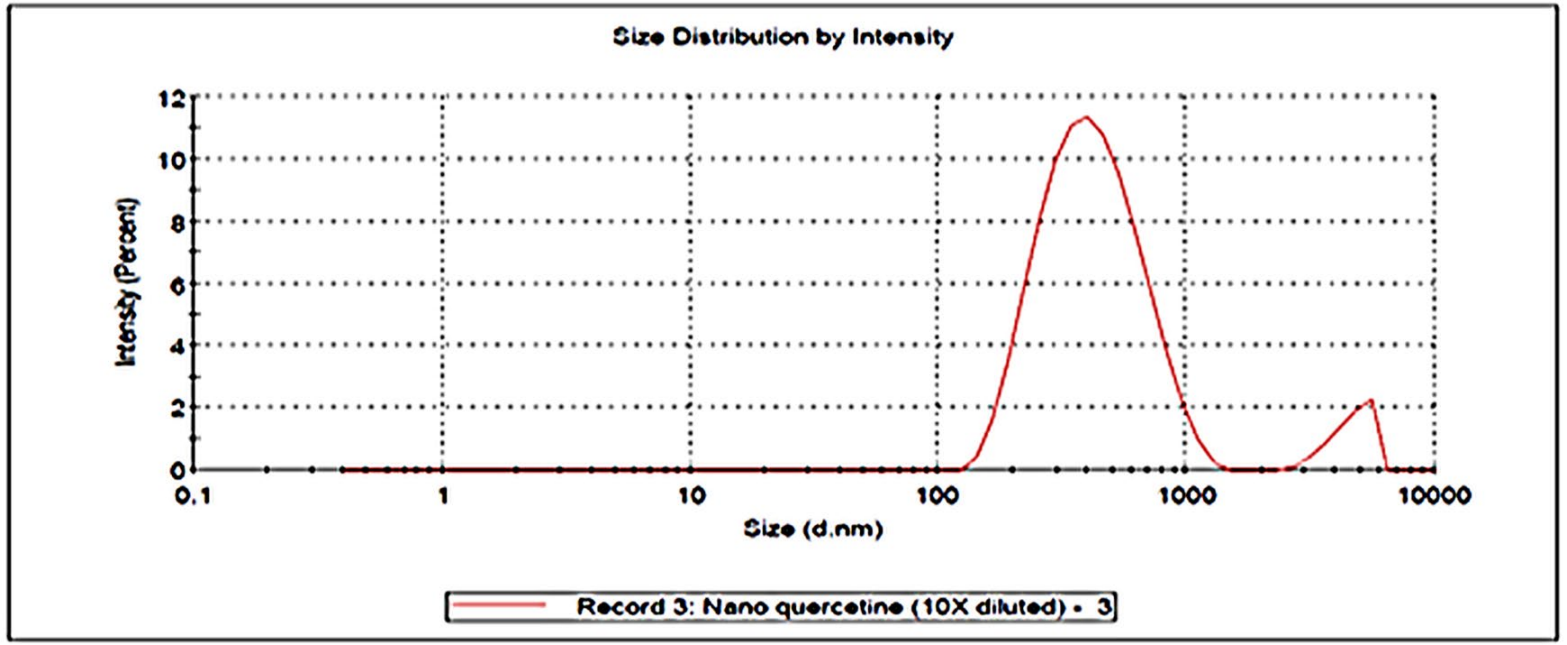
Size distribution of QCNPs by DLS.

**Fig. 2:**
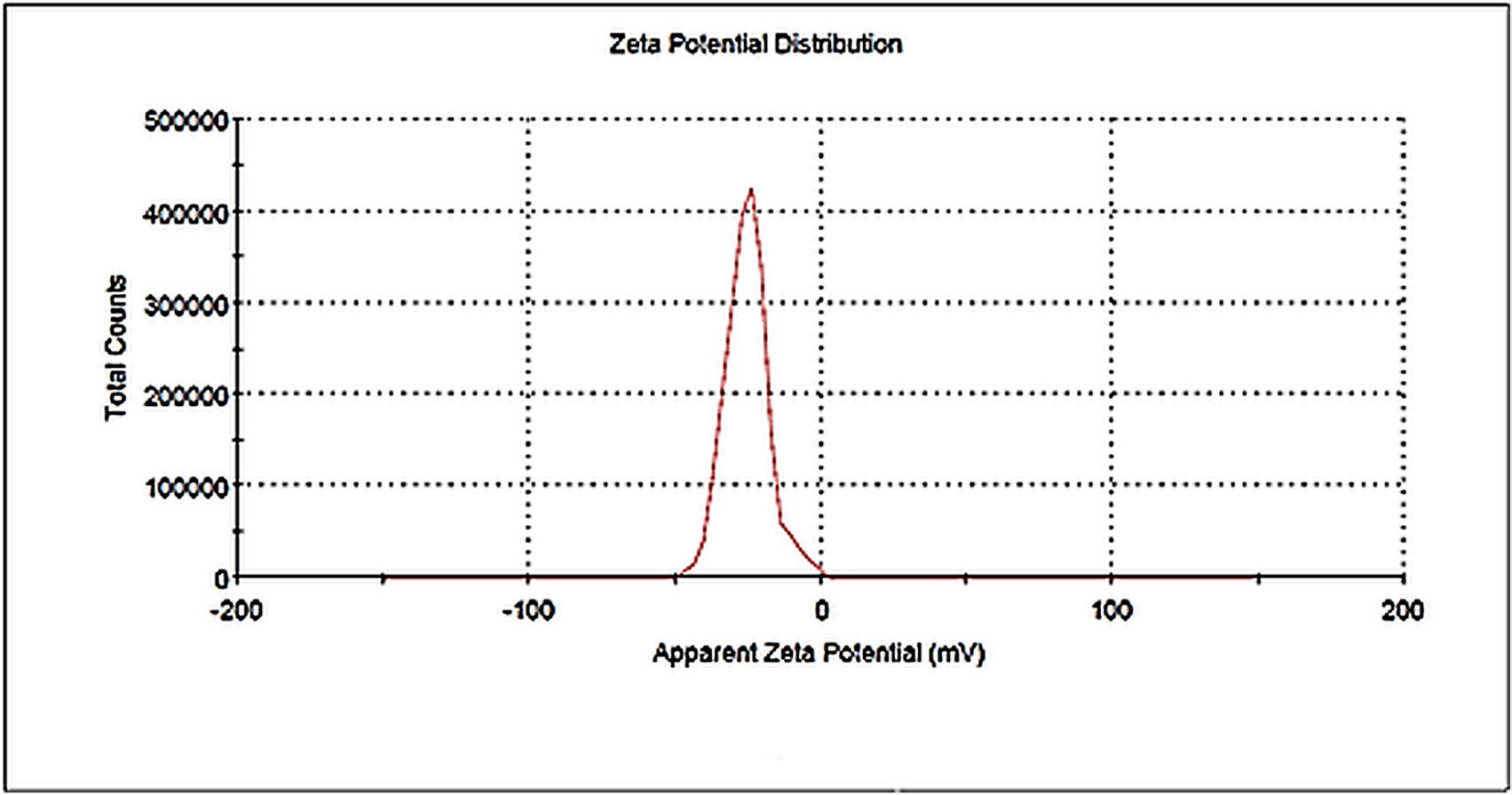
Zeta potential distribution of QCNPs.

Dynamic light scattering (DLS) analysis revealed that quercetin nanoparticles exhibited a mean hydrodynamic diameter of 405.4 ± 8.8 nm, with a polydispersity index (PDI) of 0.26 ± 0.03 (Fig. 1), indicating a moderately narrow size distribution. The zeta potential was − 23.3 ± 0.25 mV (Fig. 2), suggesting adequate colloidal stability of the nanoparticle suspension.

#### Transmission electron microscopy (TEM)

**Fig. 3:**
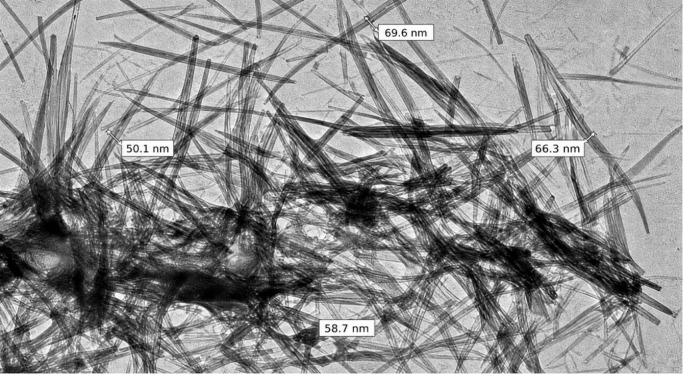
TEM image of QCNPs showing elongated needle-like morphology with nanoscale dimensions.

TEM imaging demonstrated that quercetin nanoparticles possessed a needle-like morphology, with particle dimensions ranging between 50 and 60 nm along one axis (Fig. 3), confirming successful nanoscale formation.

#### Fourier transform infrared (FTIR) spectroscopy

**Fig. 4:**
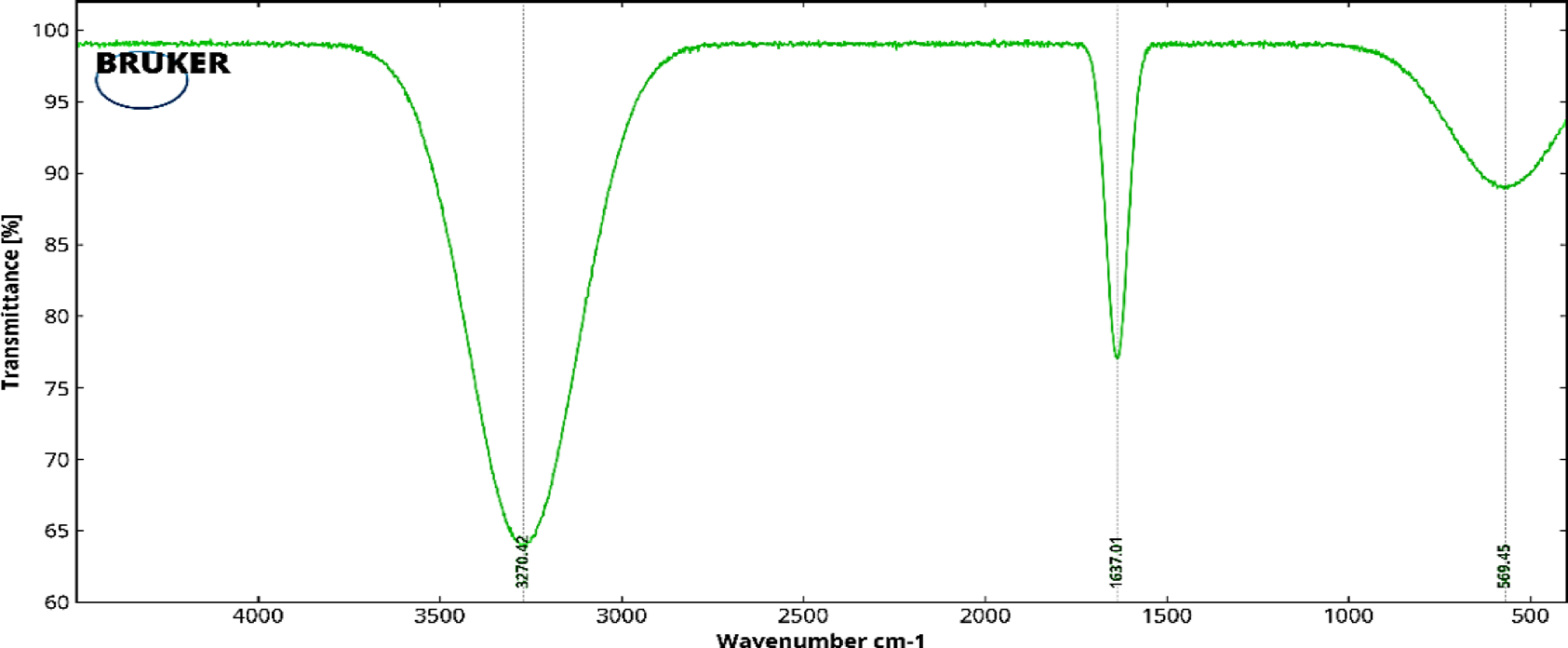
FTIR of native QC.

**Fig. 5:**
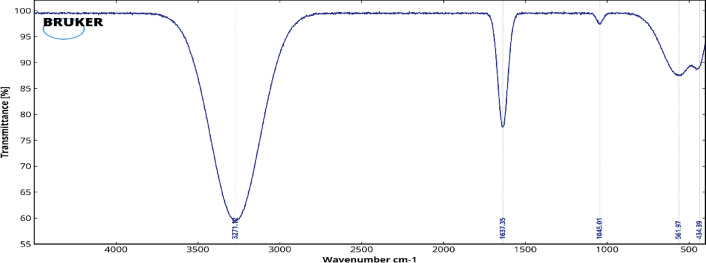
FTIR of QCNPs.

**Fig. 6:**
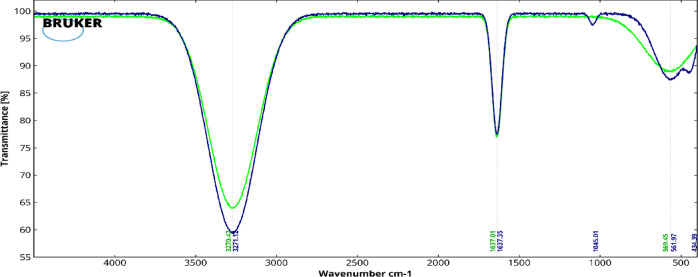
FTIR of QC with QCNPs.

FTIR spectra of quercetin nanoparticles exhibited the same characteristic absorption peaks observed in native quercetin, although with reduced peak intensities (Figs. 4, 5 and 6). This finding indicates that nanoparticle formation did not induce chemical modification or intermolecular interactions affecting the quercetin molecular structure.

### Behavioral assessments

#### Open field test

Reserpine administration resulted in a marked impairment of locomotor activity in the Parkinsonian rat model. The number of line crossings and rearing frequency decreased significantly by 97.9% and 91.1%, respectively, compared with the control group (*p* < 0.05). In contrast, time spent in the central zone and freezing duration increased significantly by 928.2% and 1389%, respectively.

Treatment with levodopa/carbidopa (L/C) partially improved locomotor performance, as evidenced by significant reductions in line crossings (− 94.6%) and rearing (− 52.5%) compared with controls. Although freezing time remained significantly elevated (+ 1041%), central zone time was restored to values comparable to the control group.

Administration of quercetin nanoparticles (QNPs) alone resulted in partial improvement of locomotor parameters; however, line crossings, rearing, and freezing time remained significantly altered relative to controls (− 63.2%, − 52.5%, and + 511%, respectively). Notably, QNPs alone or combined with L/C restored central zone time to near-control levels.

**Fig. 7:**
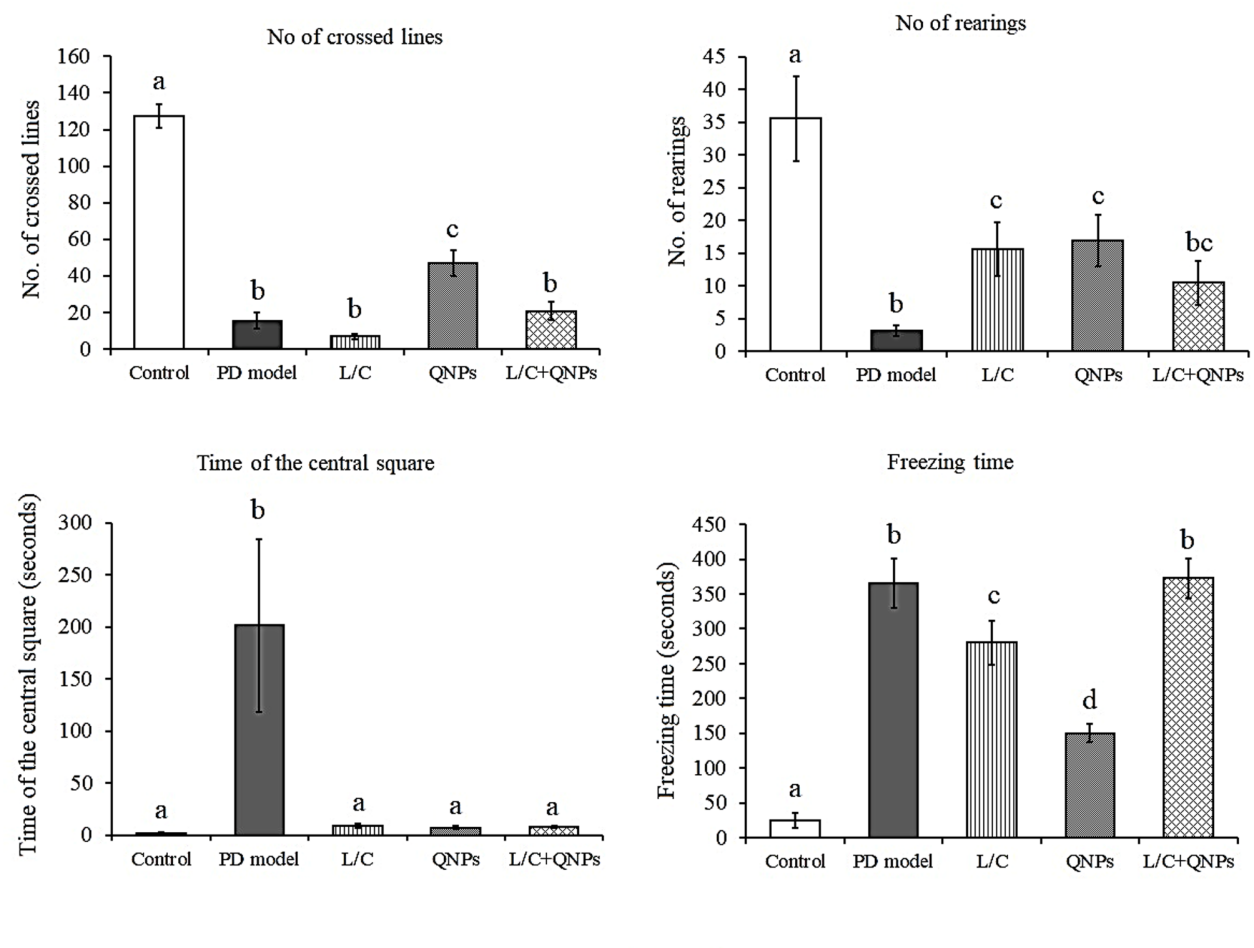
open field test results for control, PD model, PD model treated with QCNPs, and PD model treated with L-DOPA/carbidopa with QCNPs groups. The significant differences between groups were expressed by different letters, and the non-significant differences were expressed with the same letters.

Combined treatment with L/C and QNPs did not produce further significant improvements in line crossings, rearing frequency, or freezing time compared with either treatment alone, recording changes of − 83.7%, − 70.7%, and + 1419%, respectively, relative to controls (Fig. 7).

#### Grip strength (traction force) test

**Fig. 8:**
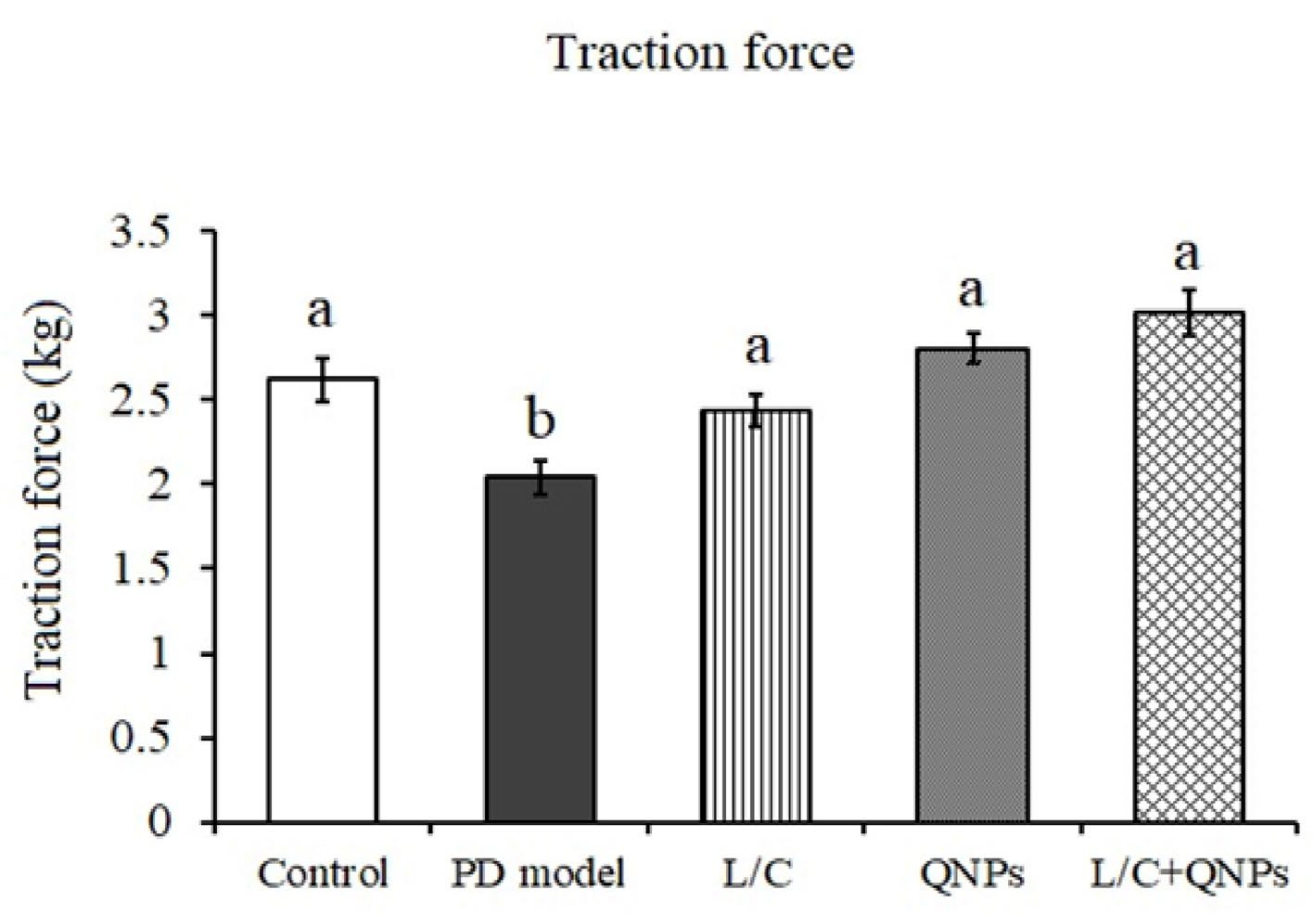
Traction force test results for control, PD model, PD model treated with QCNPs, and PD model treated with L-DOPA/carbidopa with QCNPs groups. Significant differences between groups were expressed by different letters, and the non-significant differences were expressed with the same letters.

Analysis of variance revealed a significant reduction in traction force in the PD model (− 22.1%) compared with controls (*p* < 0.05). Treatment with L/C and/or QNPs significantly restored grip strength to values comparable to the control group (Fig. 8).

### Neurochemical analyses

#### Monoamine neurotransmitters

**Fig. 9:**
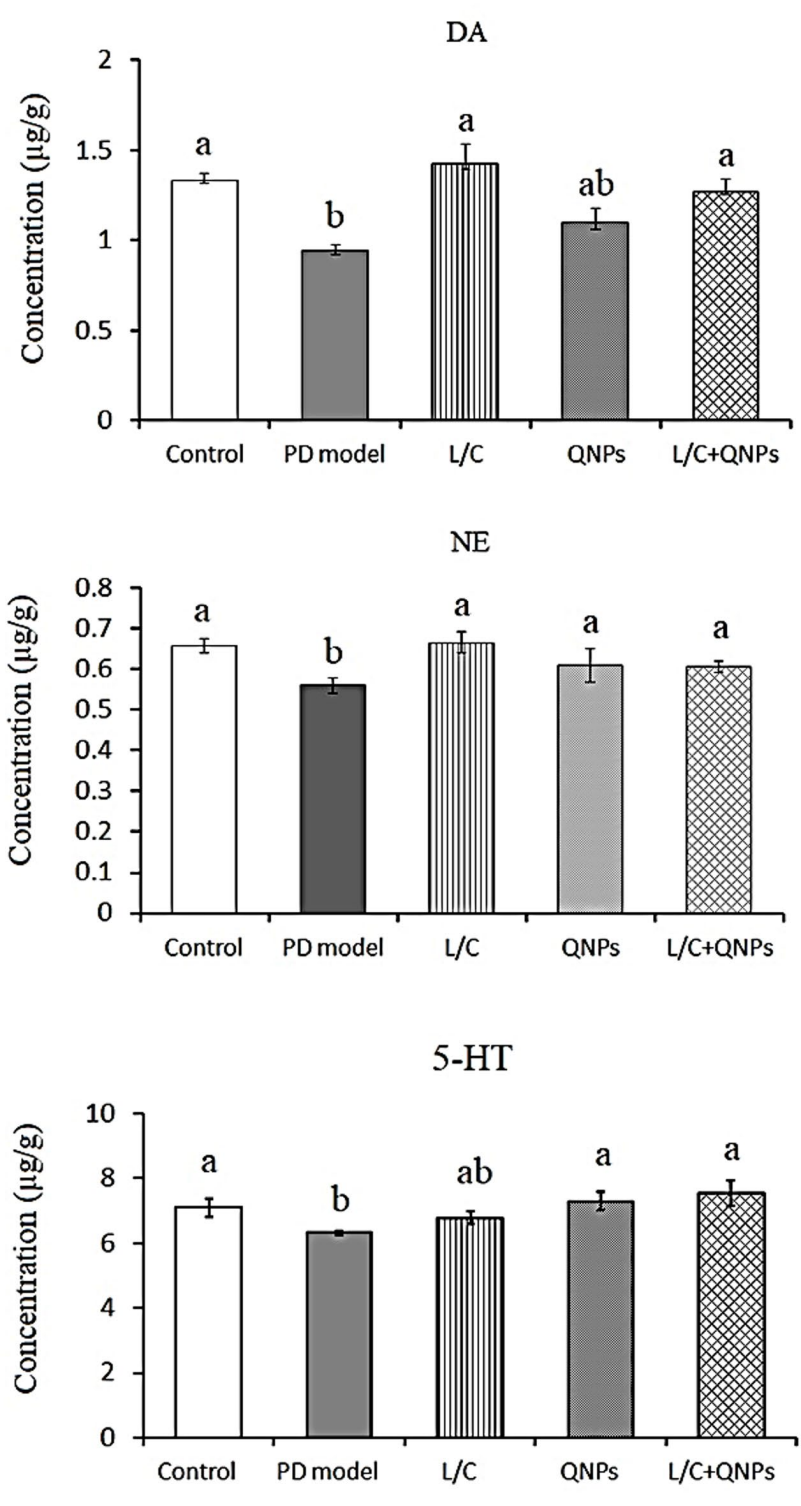
Midbrain monoamine levels for control, PD model, PD model treated with QCNPs, and PD model treated with L-DOPA/carbidopa with QCNPs groups. Significant differences between groups were expressed by different letters, and the non-significant differences were expressed with the same letters.

In the midbrain, PD rats exhibited significant reductions in dopamine (DA), norepinephrine (NE), and serotonin (5-HT) levels by 29.3%, 14.6%, and 11.0%, respectively, compared with controls (*p* = 0.001). Treatment with L/C and/or QNPs restored monoamine levels to values not significantly different from controls (Fig. 9).

**Fig. 10:**
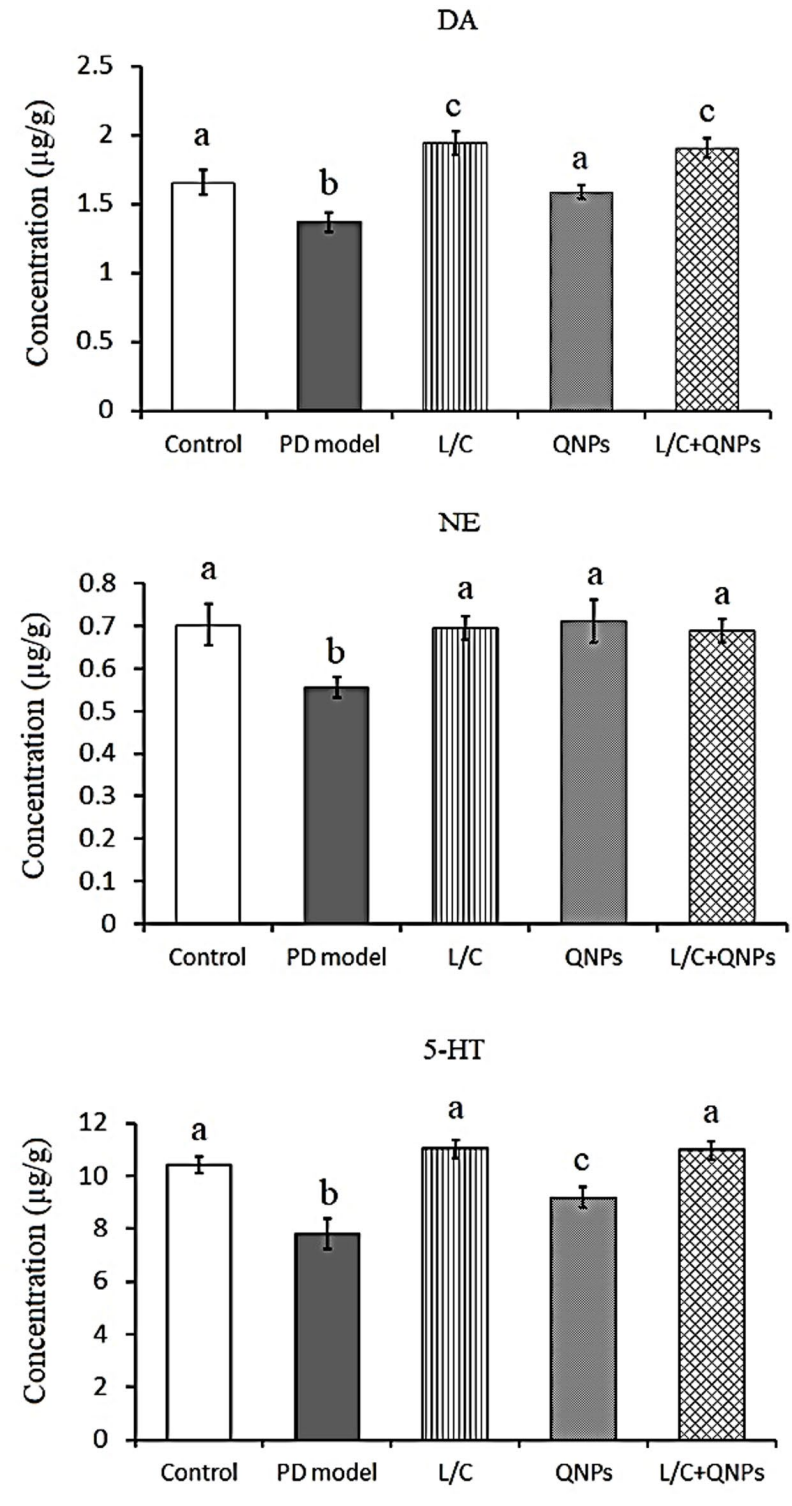
Striatum monoamine levels for control, PD model, PD model treated with QCNPs, and PD model treated with L-DOPA/carbidopa with QCNPs groups. Significant differences between groups were expressed by different letters, and the non-significant differences were expressed with the same letters.

In the striatum, DA, NE, and 5-HT levels decreased significantly by 17.3%, 20.8%, and 24.8%, respectively (*p* = 0.001). Treatment with L/C alone or combined with QNPs reversed the reduction in DA, resulting in significant increases of 17.4% and 15.1%, respectively, relative to controls. Striatal NE and 5-HT levels were restored to near-control values following L/C treatment alone or in combination with QNPs. Treatment with QNPs alone restored striatal DA and NE levels to near-control values; however, 5-HT levels remained significantly reduced (− 11.8%) compared with controls (Fig. 10).

#### Acetylcholinesterase (AChE) and monoamine oxidase (MAO) activities

**Fig. 11:**
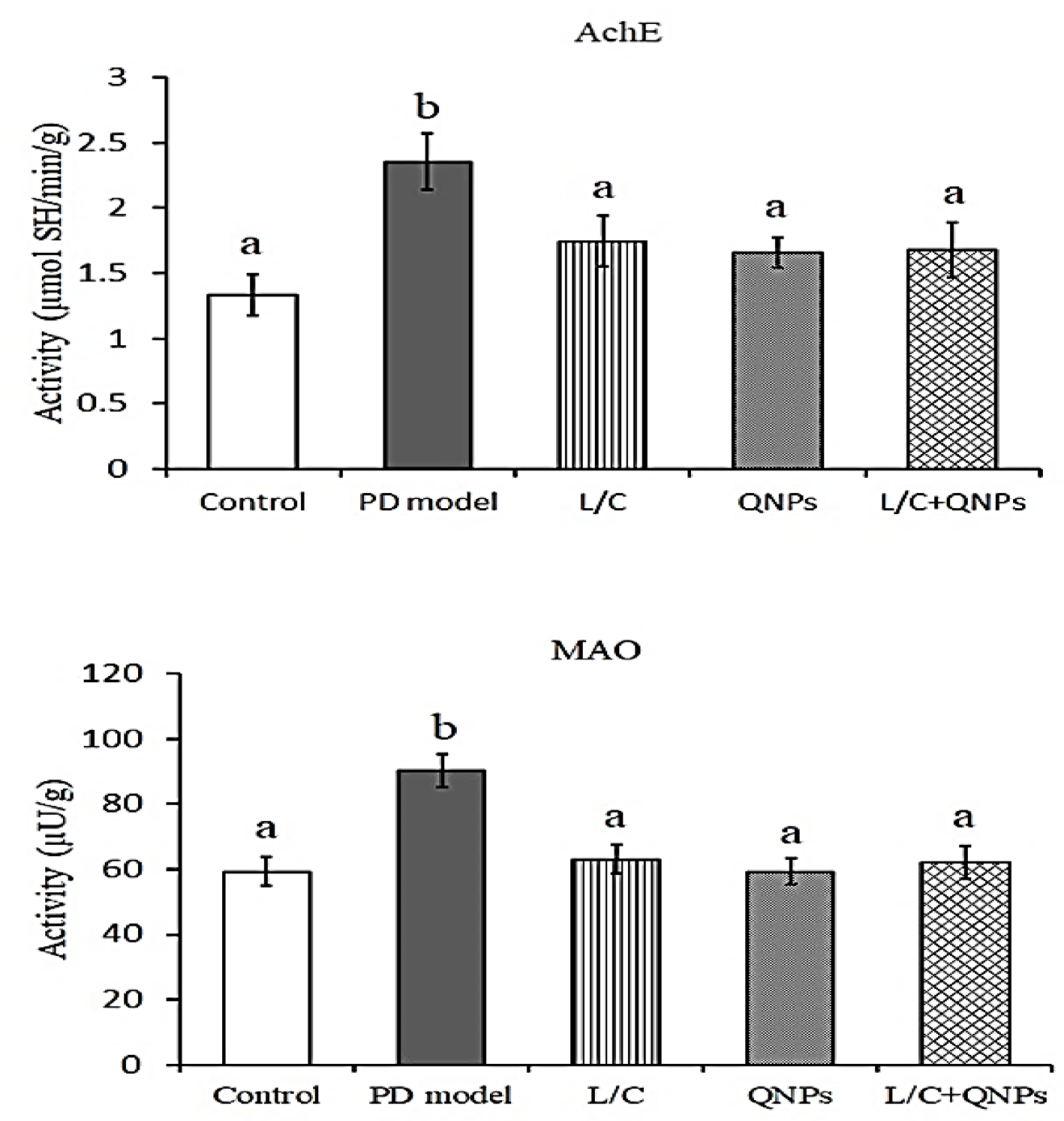
Midbrain enzyme activities for control, PD model, PD model treated with QCNPs, and PD model treated with L-DOPA/carbidopa with QCNPs groups. Significant differences between groups were expressed by different letters, and the non-significant difference was expressed with the same letters.

Significant alterations in AChE and MAO activities were observed in both the midbrain and striatum of PD rats (*p* = 0.001). In the midbrain, AChE and MAO activities increased by 76.9% and 52.1%, respectively, compared with controls. Treatment with L/C and/or QNPs normalized these enzyme activities to control-like levels (Fig. 11).

**Fig. 12:**
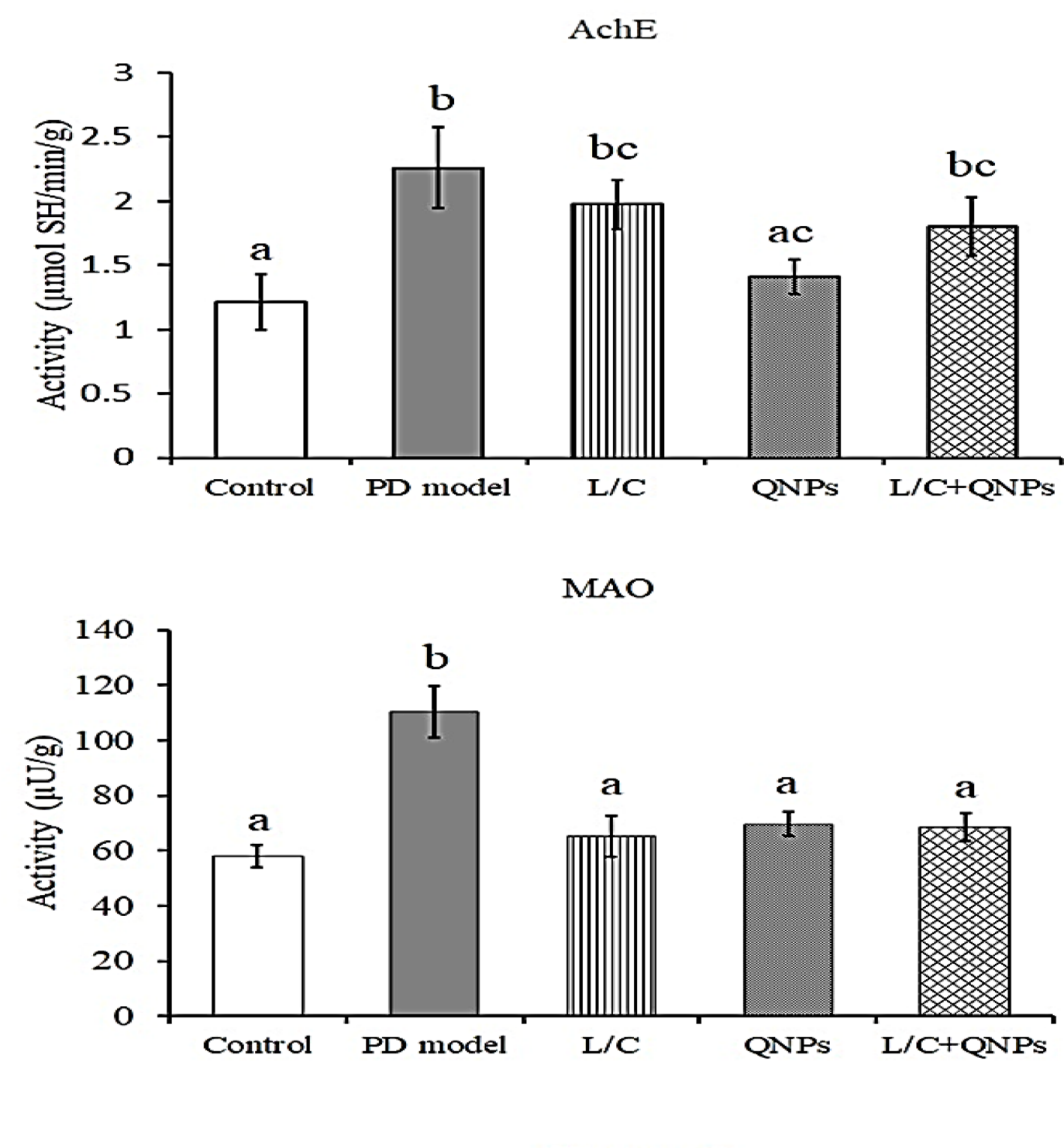
Striatal enzyme activities for control, PD model, PD model treated with QCNPs, and PD model treated with L-DOPA/carbidopa with QCNPs groups. Significant differences between groups were expressed by different letters, and the non-significant differences were expressed with the same letters.

In the striatum, AChE and MAO activities increased significantly by 86.4% and 90.1%, respectively. Treatment with QNPs restored striatal AChE activity to near-control values, whereas L/C alone failed to fully reverse the elevation. In contrast, MAO activity was restored to near-control levels following treatment with L/C and/or QNPs (Fig. 12).

#### Oxidative stress markers

**Fig. 13:**
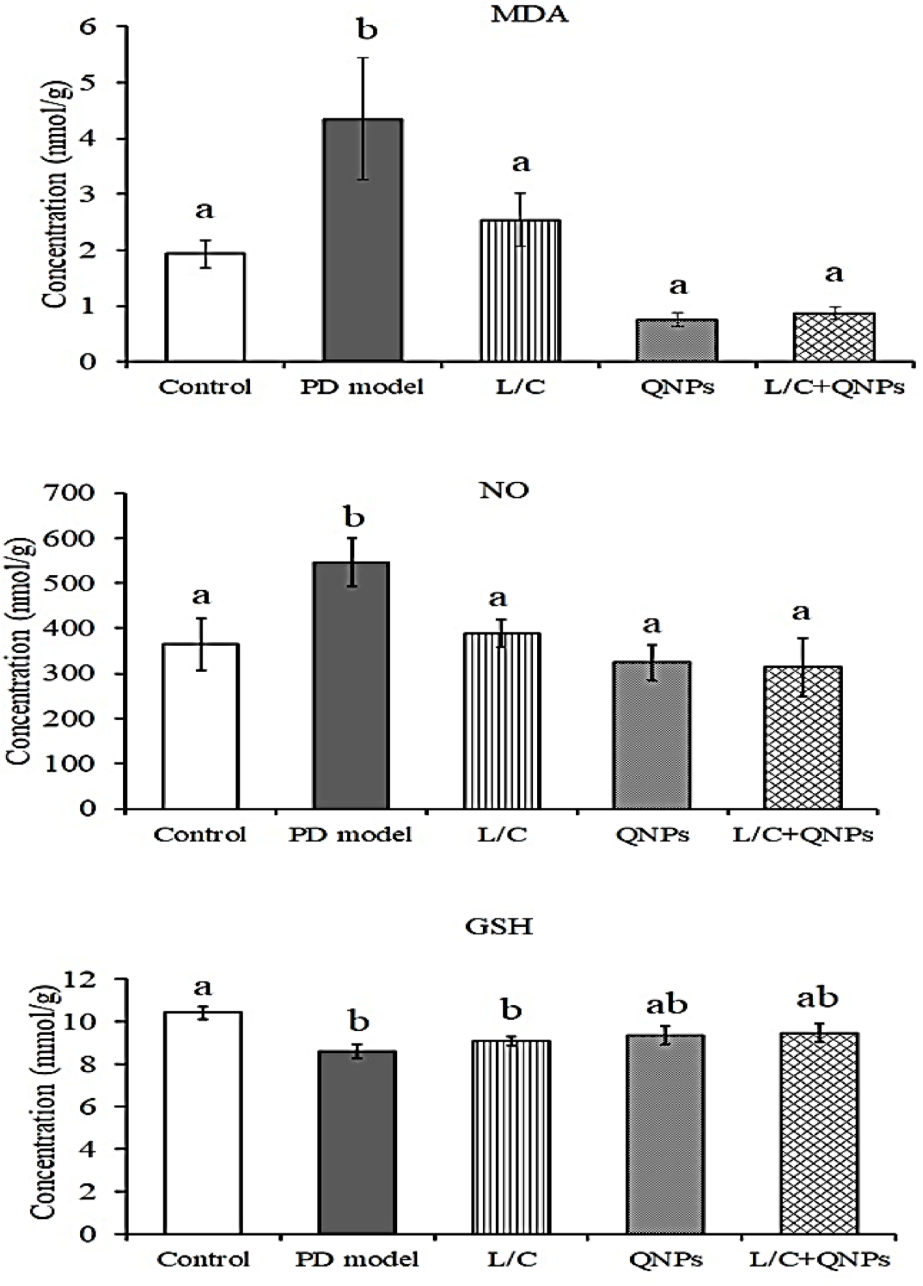
Midbrain oxidative stress biomarkers in control, PD model, PD model treated with QCNPs, and PD model treated with L-DOPA/carbidopa with QCNPs groups. Significant differences between groups were expressed by different letters, and the non-significant differences were expressed with the same letters.

In the midbrain, PD rats exhibited significant increases in malondialdehyde (MDA; + 125.6%, *p* = 0.001) and nitric oxide (NO; +50.1%, *p* = 0.015), along with a significant reduction in reduced glutathione (GSH; − 17.7%, *p* = 0.023). Treatment with L/C normalized MDA and NO levels but did not fully restore GSH levels ( − 12.8%). In contrast, QNPs alone or in combination with L/C effectively prevented oxidative stress and restored GSH to near-control values (Fig. 13).

**Fig. 14:**
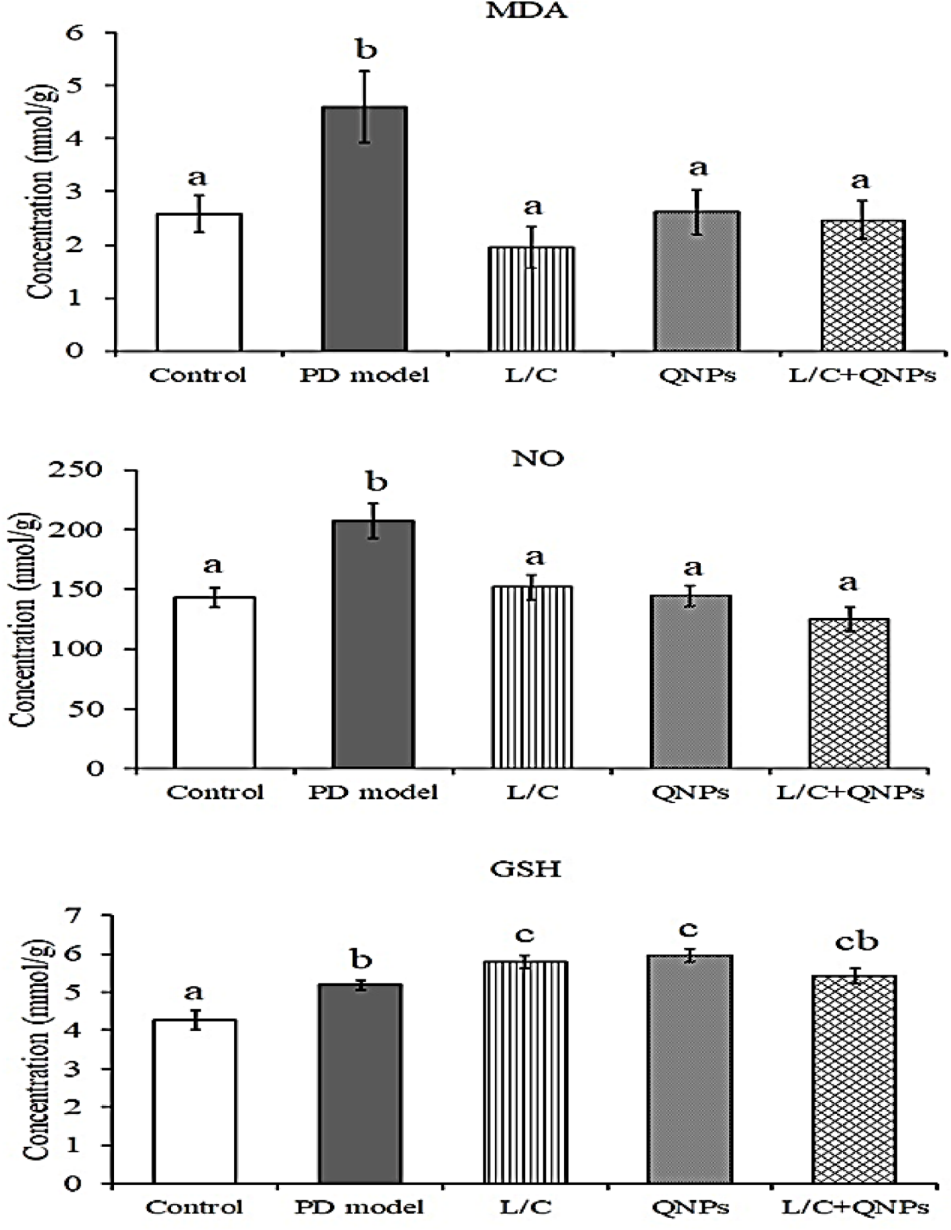
Striatal oxidative stress biomarkers in control, PD model, PD model treated with QCNPs, and PD model treated with L-DOPA/carbidopa with QCNPs groups. Significant differences between groups were expressed by different letters, and the non-significant differences were expressed with the same letters.

In the striatum, MDA (+ 78.2%, *p* = 0.002), NO (+ 44.8%, *p* = 0.001), and GSH (+ 21.8%, *p* = 0.001) levels were significantly elevated in PD rats. Treatment with L/C and/or QNPs restored MDA and NO levels to near-control values. Striatal GSH levels remained significantly elevated following L/C or QNPs treatment, with the combined treatment producing a comparable increase (Fig. 14).

#### BDNF and TNF-α levels

**Fig. 15:**
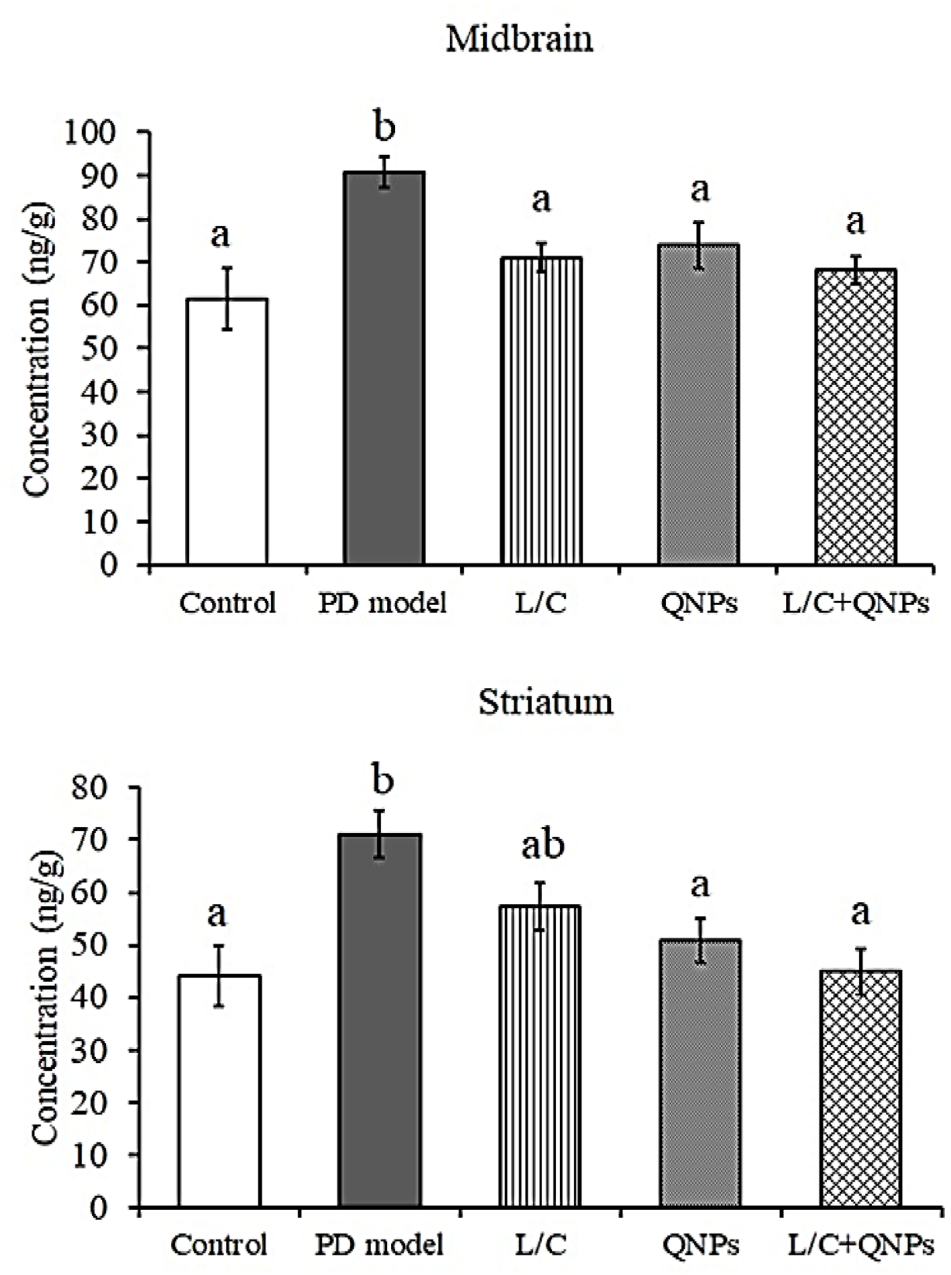
BDNF levels in both midbrain and striatum regions in the control, PD model, PD model treated with QCNPs, and PD model treated with L-DOPA/carbidopa with QCNPs groups. The significant differences between groups were expressed by different letters, and the non-significant differences were expressed with the same letters.

PD induction resulted in a significant increase in BDNF levels in both the midbrain (+ 47.6%, *p* = 0.004) and striatum (+ 61.4%, *p* = 0.002). Treatment with L/C and/or QNPs restored BDNF levels to values not significantly different from controls (Fig. 15).

**Fig. 16:**
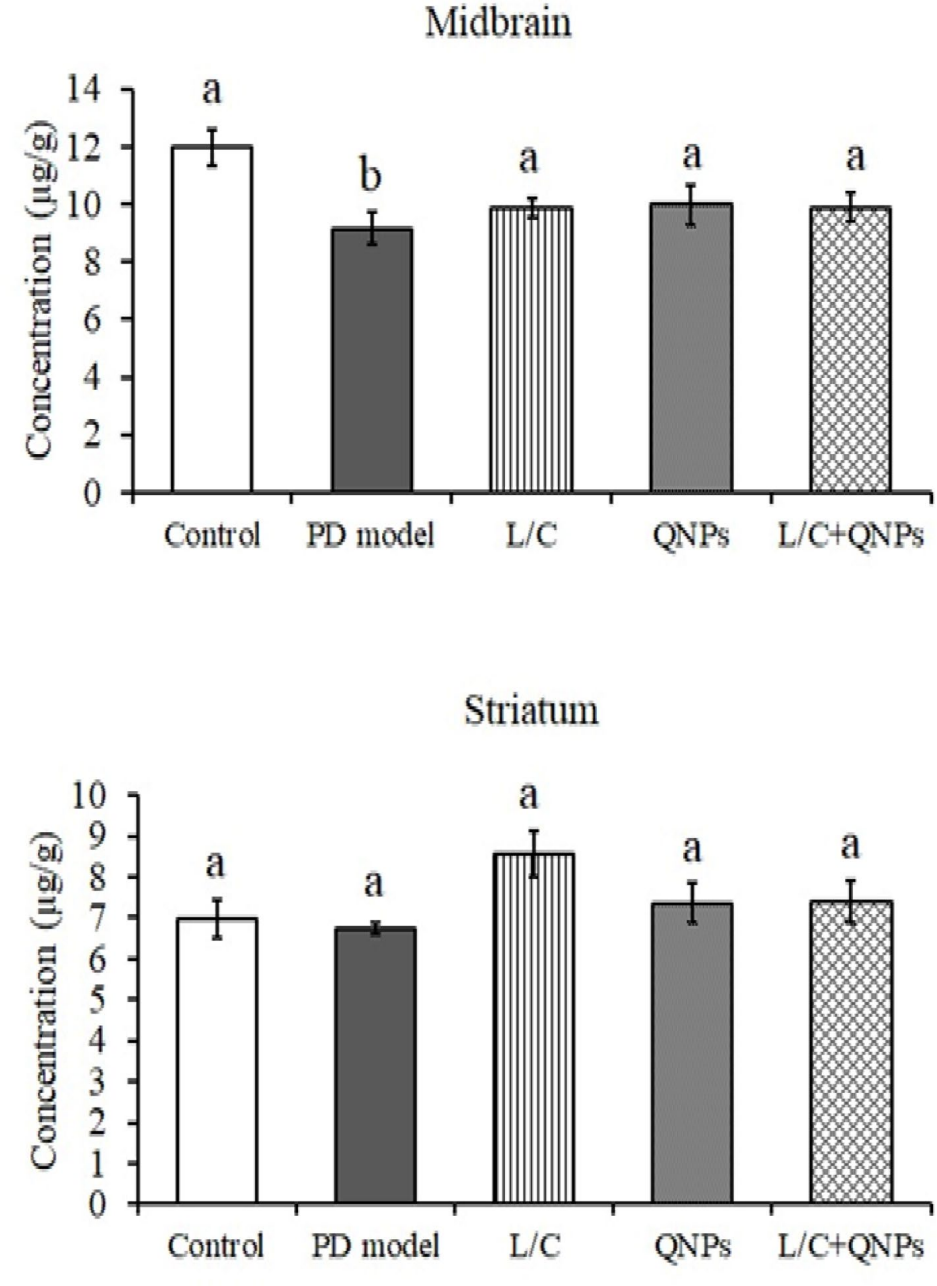
TNF-α levels in both midbrain and striatum regions in the control, PD model, PD model treated with QCNPs, and PD model treated with L-DOPA/carbidopa with QCNPs groups. Significant differences between groups were expressed by different letters, and the non-significant differences were expressed with the same letters.

In the midbrain, TNF-α levels were significantly reduced (− 23.6%, *p* = 0.025) in PD rats, whereas treatment with L/C and/or QNPs restored TNF-α levels to near-control values. No significant changes in TNF-α levels were observed in the striatum across experimental groups (Fig. 16).

## Discussion

Parkinson’s disease (PD) is a progressive neurodegenerative disorder in which oxidative and nitrosative stress play central roles in dopaminergic neuronal loss. Excessive generation of reactive oxygen species (ROS) and reactive nitrogen species (RNS), coupled with impaired antioxidant defenses, leads to lipid, protein, and DNA damage, ultimately resulting in neuronal degeneration^28^.

In the present study, a significant increase in lipid peroxidation, as reflected by elevated malondialdehyde (MDA) levels, was observed in both the midbrain and striatum of the reserpine-induced PD rat model. MDA is a well-established marker of oxidative stress–mediated membrane damage and neuronal injury^29^. Enhanced lipid peroxidation has been linked to α-synuclein misfolding, which accelerates dopaminergic neuronal degeneration in PD^30^. The vulnerability of the brain to oxidative stress is well documented and is attributed to its high lipid content, elevated oxygen consumption, and limited antioxidant capacity^31^.

In parallel, nitric oxide (NO) levels were significantly elevated in the midbrain and striatum of PD animals. Excess NO can react with superoxide radicals to form peroxynitrite, a highly cytotoxic species that induces nitration of proteins, DNA damage, and mitochondrial dysfunction, thereby promoting neuronal apoptosis^32^. These findings confirm that reserpine induces pronounced oxidative and nitrosative stress in nigrostriatal regions.

Reduced glutathione (GSH), a critical non-enzymatic antioxidant in the brain, was significantly depleted in the midbrain but increased in the striatum. GSH depletion has been implicated in the early stages of PD and is associated with enhanced neuroinflammation and neuronal vulnerability^33^. The regional differences observed in the present study may reflect differential susceptibility to oxidative stress. The midbrain, which contains the cell bodies of dopaminergic neurons in the substantia nigra, appears more vulnerable to oxidative damage than the striatum, which predominantly contains dopaminergic terminals. The elevated striatal GSH level may therefore represent a compensatory antioxidant response.

Reserpine administration resulted in a marked depletion of dopamine (DA) in both the midbrain and striatum, consistent with its inhibitory effect on vesicular monoamine transporter-2 (VMAT2), leading to impaired monoamine storage and enhanced cytosolic degradation (Fernandes et al., 2012). Dopamine depletion is the principal pathological hallmark of PD and directly underlies motor impairments such as bradykinesia, rigidity, postural instability, and tremors, as well as non-motor manifestations including cognitive decline, sleep disturbances, and depression^34^. In addition, the observed reductions in norepinephrine (NE) and serotonin (5-HT) may contribute to non-motor symptoms such as mood disorders and insomnia commonly associated with PD^35^.

The significant increase in monoamine oxidase (MAO) activity observed in the midbrain and striatum further explains the depletion of monoamine neurotransmitters. MAO-mediated oxidative catabolism of monoamines generates hydrogen peroxide and other ROS, thereby exacerbating oxidative stress^36^. Thus, elevated MAO activity represents both a cause and a consequence of monoamine depletion and oxidative injury in the PD model.

Acetylcholinesterase (AChE) activity was also significantly increased in both brain regions, indicating enhanced cholinergic tone. The balance between dopaminergic and cholinergic signaling within the striatum is critical for normal motor function^37^. Loss of dopaminergic input disrupts this balance, resulting in excessive cholinergic activity that contributes to motor dysfunction. This imbalance is reflected behaviorally by impaired locomotor activity and reduced traction force observed in the present study. The combination of locomotor and muscular assessments used in the present work is consistent with previous studies validating the reserpine-induced Parkinsonian phenotype^19^. Conversely, the increased cholinergic activity in the midbrain may represent a compensatory mechanism aimed at stimulating residual dopaminergic neurons through nicotinic receptor activation^38^.

Brain-derived neurotrophic factor (BDNF) levels were significantly elevated in both the midbrain and striatum of PD rats. BDNF is a key neurotrophin involved in neuronal survival, plasticity, and repair^39^. Its upregulation likely reflects an endogenous neuroprotective response to dopaminergic injury, consistent with previous reports in neurodegenerative conditions^40,41^. BDNF signaling through TrkB and p75^NTR^ receptors has been implicated in synaptic remodeling and neuronal recovery^42^.

The role of tumor necrosis factor-α (TNF-α) in PD remains complex and context-dependent. In the present study, TNF-α levels were significantly reduced in the midbrain but remained unchanged in the striatum. Previous studies have reported both neurotoxic and neuroprotective roles for TNF-α, depending on the timing and extent of its expression^43,44^. TNF-α may exert indirect neuroprotective effects by modulating insulin-like growth factor-1 (IGF-1) expression, which supports dopaminergic neuron survival^45^.

Quercetin is a potent flavonoid antioxidant capable of crossing the blood–brain barrier. However, its clinical utility is limited by poor solubility and low bioavailability^46^. In the present study, nano-formulated quercetin (QNPs) was employed to overcome these limitations. QNPs effectively attenuated oxidative and nitrosative stress by reducing MDA and NO levels and restoring GSH content in both brain regions. These effects may be attributed to the intrinsic antioxidant properties of quercetin and its ability to suppress MAO activity, as previously reported^47^.

Importantly, QNPs restored dopamine levels in the striatum and significantly improved monoamine neurotransmitter balance in the midbrain and striatum. The normalization of AChE activity further indicates restoration of dopaminergic–cholinergic equilibrium, which translated into significant behavioral improvement in locomotor activity and muscle strength. These findings highlight the therapeutic potential of QNPs in mitigating both neurochemical and behavioral deficits in PD.

Treatment with levodopa/carbidopa (L/C) produced the expected restoration of monoamine levels and reduction in MAO activity, thereby alleviating oxidative stress and motor impairment. However, the supraphysiological increase in striatal dopamine observed with L/C treatment underscores known limitations associated with dopaminergic replacement therapy. Notably, combined administration of QNPs and L/C did not produce synergistic benefits and, in some parameters, suggested possible antagonistic interactions, particularly with respect to AChE activity and freezing behavior. This may indicate that both interventions partially converge on overlapping pathways related to oxidative stress modulation and dopaminergic preservation. Moreover, levodopa primarily addresses symptomatic dopamine replacement, whereas quercetin nanoparticles may exert broader antioxidant and anti-inflammatory effects. Future studies should include detailed pharmaceutical characterization, including release kinetics, storage stability, encapsulation efficiency, pharmacokinetics, and bioavailability analyses.

In conclusion, the present study demonstrates that nano-quercetin exerts a robust neuroprotective effect against reserpine-induced Parkinsonian pathology. Its efficacy is comparable to that of standard levodopa/carbidopa therapy and is mediated through antioxidant, anti-nitrosative, MAO-inhibitory, and neurotransmitter-restorative mechanisms. These findings support the potential of QNPs as a promising adjunct or alternative therapeutic strategy for Parkinson’s disease.

## Data Availability

The datasets generated and/or analyzed during the current study are available from the corresponding author upon reasonable request.
